# Detection of Motor Cerebral Activity After Median Nerve Stimulation During General Anesthesia (STIM-MOTANA): Protocol for a Prospective Interventional Study

**DOI:** 10.2196/43870

**Published:** 2023-02-02

**Authors:** Sébastien Rimbert, Julien Lelarge, Philippe Guerci, Seyed Javad Bidgoli, Claude Meistelman, Guy Cheron, Ana Maria Cebolla Alvarez, Denis Schmartz

**Affiliations:** 1 CHU Brugmann Université Libre de Bruxelles Bruxelles Belgium; 2 Laboratory of Neurophysiology and Movement Biomechanics Université Libre de Bruxelles Neurosciences Institute Bruxelles Belgium; 3 Inria Bordeaux Sud-Ouest University of Bordeaux Talence France; 4 Department of Anesthesiology and Critical Care Medicine University Hospital of Nancy Vandoeuvre-lès-Nancy France

**Keywords:** intraoperative awareness, brain-computer interfaces, median nerve stimulation, electroencephalography, general anesthesia

## Abstract

**Background:**

Accidental awareness during general anesthesia (AAGA) is defined as an unexpected awareness of the patient during general anesthesia. This phenomenon occurs in 1%-2% of high-risk practice patients and can cause physical suffering and psychological after-effects, called posttraumatic stress disorder. In fact, no monitoring techniques are satisfactory enough to effectively prevent AAGA; therefore, new alternatives are needed. Because the first reflex for a patient during an AAGA is to move, but cannot do so because of the neuromuscular blockers, we believe that it is possible to design a brain-computer interface (BCI) based on the detection of movement intention to warn the anesthetist. To do this, we propose to describe and detect the changes in terms of motor cortex oscillations during general anesthesia with propofol, while a median nerve stimulation is performed. We believe that our results could enable the design of a BCI based on median nerve stimulation, which could prevent AAGA.

**Objective:**

To our knowledge, no published studies have investigated the detection of electroencephalographic (EEG) patterns in relation to peripheral nerve stimulation over the sensorimotor cortex during general anesthesia. The main objective of this study is to describe the changes in terms of event-related desynchronization and event-related synchronization modulations, in the EEG signal over the motor cortex during general anesthesia with propofol while a median nerve stimulation is performed.

**Methods:**

STIM-MOTANA is an interventional and prospective study conducted with patients scheduled for surgery under general anesthesia, involving EEG measurements and median nerve stimulation at two different times: (1) when the patient is awake before surgery (2) and under general anesthesia. A total of 30 patients will receive surgery under complete intravenous anesthesia with a target-controlled infusion pump of propofol.

**Results:**

The changes in event-related desynchronization and event-related synchronization during median nerve stimulation according to the various propofol concentrations for 30 patients will be analyzed. In addition, we will apply 4 different offline machine learning algorithms to detect the median nerve stimulation at the cerebral level. Recruitment began in December 2022. Data collection is expected to conclude in June 2024.

**Conclusions:**

STIM-MOTANA will be the first protocol to investigate median nerve stimulation cerebral motor effect during general anesthesia for the detection of intraoperative awareness. Based on strong practical and theoretical scientific reasoning from our previous studies, our innovative median nerve stimulation–based BCI would provide a way to detect intraoperative awareness during general anesthesia.

**Trial Registration:**

Clinicaltrials.gov NCT05272202; https://clinicaltrials.gov/ct2/show/NCT05272202

**International Registered Report Identifier (IRRID):**

PRR1-10.2196/43870

## Introduction

Unexpectedly awakening during surgery is a terrifying experience for patients, who fear it, but also for the medical staff, who are worried that the situation may occur under their supervision [1]. This type of phenomenon is called an accidental awareness during general anesthesia (AAGA) [2]. Although its origin is still debated, the reasons and factors favoring AAGA are being studied. This phenomenon appears if the depth of anesthesia, induced by the concentration of anesthetic used, is not deep enough to compensate for all the surgical stimuli related to the operation in progress. The role of the anesthetist is to succeed in inducing an altered state of consciousness, which is complexly intertwined with analgesia and amnesia. Indeed, if the anesthesia is too light, it may result in recovery of consciousness and volitional movement during the operation, which would be very dangerous for the patient [3]. Conversely, if too much anesthetic agent is given, it increases the depth of anesthesia but may lead to problems such as adverse cardiovascular effects, postoperative delirium, and cognitive impairment, increasing the probability of mortality during surgery [4]. Finally, an anesthetic overdose may also increase the symptoms of postoperative nausea and vomiting and thus delay the patient’s discharge from the hospital [5].

The rate of AAGA in high-risk practices (eg, obstetric, cardiac, and thoracic) varies from 1% to2% [6]. Considering that hundreds of millions of general anesthesia are performed each year worldwide, new solutions to better prevent this phenomenon are needed [7,8]. AAGA can also cause physical pain [2] and lead to psychological sequelae called posttraumatic stress disorder [1-3], which can last for several years and have severe psychological effects leading to suicide [9,10]. From the economic impact perspective, AAGAs generate direct costs because it is one of the top 3 causes of complaints against hospitals (*>*US $43,000 per claim) [2,3,11] and indirect costs from the expenses related to the follow-up and psychological support of the patient who has experienced an AAGA. There are currently 2 procedures to monitor the depth of anesthesia based either on the observation of the clinical features (eg, heart rate, temperature, blood pressure, and movement) or electroencephalographic (EEG) analysis. Unfortunately, the first one does not prevent AAGA during surgery may be because such clinical signs observations remain a very indirect way of monitoring the patients’ cerebral state not always enabling the anesthesiologist to predict an AAGA before it occurs [12]. Although new devices based on frontal EEG activity indexes are already in use, the studies demonstrating the superiority compared to clinical surveillance or end-tidal anesthetic gas [13-16] are not conclusive. Moreover, several studies consent to the use of BIS as it has been shown that the predictive accuracy is decreased when using neuromuscular blockers [1].

The patients’ testimonies show that the first reaction during an AAGA is usually to move to alert the medical staff [2,17]. Unfortunately, during most surgeries, the patient is curarized, which causes neuromuscular block and prevents any movement. Detecting movement intention (MI) is possible by analyzing EEG signals from the motor cortex with a brain-computer interface (BCI). This is based on previous evidence demonstrating that simple MIs are characterized by power spectrum variations (ie, event-related (de)-synchronization called ERD/ERS) in the EEG mu (7-13 Hz) and beta (15-35 Hz) bands over the motor cortex [18] and that electrical stimulation of the median nerve also induces changes in the cortical activity [19,20], which are visible in the EEG signal and which are similar to an MI [21,22]. Interestingly, our preliminary results suggested that when a hand MI coincides with median nerve stimulation (MNS), the ERD/ERS is considerably reduced, and thus the MI can be detected much better by machine learning techniques than an MI without MNS [23-26]. The originality of this BCI paradigm is to exploit this MNS-induced phenomenon to accurately detect the patient’s motor intention during an AAGA. Based on these previous results, we envisage a routine procedure where the anesthetized patient is stimulated by electrical pulses delivered at the median nerve position at the wrist, while a BCI device analyzes the ERD and ERS modulations over the motor cortex to detect the intention of movement of the patient. Our previous clinical trial (MOTANA protocol [23]) also includes a condition with MNS and another with MNS accompanied by MI for 3 concentrations of propofol (0, 0.5 µg/mL, and 1 µg/mL). The preliminary results (n=15) highlighted the advantage of a BCI based on MNS from both a functional and an accuracy point of view. First, MNS intrinsically provides a trigger to know when to analyze the signal. Second, the classification accuracy of an MNS-based BCI appeared to be way better than that of a BCI aiming to discriminate a motor imagination from a resting state. More precisely, an MNS-based BCI detected the MI state 15% more accurately. For several subjects, the use of MNS improves performance by more than 20% compared to a standard method [22,25].

These preliminary results also showed that propofol sedation (at 0.5 µg/mL and 1 µg/mL) has no negative impact on the ability of an MNS-based BCI to detect MI. Concretely, at relatively low concentrations, ERD/ERS patterns are still present in the sensorimotor cortex [26]. From these encouraging results, we intend now to verify that the oscillatory activity generated by the MNS remains present during general anesthesia with higher concentrations of propofol. This is in line with the existing literature, suggesting that the detection of MNS under general anesthesia can be achieved as somatosensory cortex maintains a certain activity confirmed by the presence of evoked potentials during sleep or general anesthesia-induced unconsciousness [27]. The cortical regions may still be receptive to information, but their ability to communicate with other regions may be altered during general anesthesia [28]. According to our knowledge, no study has investigated the way in which the cerebral motor activity was modulated with peripheral nerve stimulation over the sensorimotor cortex during general anesthesia. The aim of this study is to verify that ERD/ERS following MNS is still present and detectable under high doses of propofol with the EEG technique. Such new knowledge will enable the design of an innovative BCI specialized in the detection of intraoperative awareness during general anesthesia.

The first objective of this study is to verify that ERD and ERS patterns induced by MNS can be detected in the cortical motor EEG signal under various concentrations of propofol corresponding to the anesthetic doses required for general anesthesia. The secondary objective is to characterize and understand how the ERD and ERS generated after each MNS will be altered according to each concentration of propofol at the effect site. These results will be discussed in relation to previous results obtained from a protocol investigating the effect of light propofol sedation on EEG signals in the motor cortex. The third objective is to analyze EEG signals offline and try to detect MNS under propofol with new machine learning algorithms. Complementary, the short and middle latency components of the somatosensory evoked potentials (SEPs) will be analyzed for different propofol concentrations. Finally, the long-term goal is to design a BCI that can detect intraoperative awareness during general anesthesia.

## Methods

### Study Design

The study will be held in an approved location in the operating theaters, at the University Hospital of Bruxelles-Brugmann (Belgium). An anesthetic evaluation will be performed by the study investigator (DS) within 1 month prior to surgery. During this consultation, the study protocol will be explained to the patient, and the different explanatory documents will be given. Only eligible subjects will be enrolled (inclusion and exclusion criteria are listed). Participation is completely voluntary and withdrawal will be always possible at any time without affecting the level of care that will be received. There will be no financial compensation for this study. Each voluntary patient recruited for the study will benefit from the standard of care before, during, and after the surgery. On the day of surgery, and as recommended, the anesthetist will check that there are no current contraindications to perform general anesthesia (ie, failure to fast, fever, occurrence of other medical conditions or disease, unusual blood sample, etc).

Recording a patient's baseline EEG during MNS without an anesthetic drug administered is a prerequisite for the proper functioning of the BCI. It will take place before surgery in a specific quiet room in the postanesthesia care unit. The estimated time to set the EEG headset is 25 minutes, and the estimated time for baseline EEG monitoring is 20 minutes (see Table 1). Painless discontinuous stimulation of the median nerve and recording of the patient's EEG signal will take place throughout the operation. The anesthesia protocol will be left at the discretion of the anesthesiologist, except for obtaining hypnosis, which will have to be performed with propofol using a target-controlled infusion (TCI) pump with the Schnider pharmacokinetic model at the effect site [29-31]. If necessary, neuromuscular blocker agents will be used and monitoring will be performed. Data collection of the propofol site effect target administered to the patient will be directly recorded on a computer. After the surgery is completed, sedation will be discontinued allowing the patient to recover and be monitored afterward in the postanesthesia care unit.

**Table 1:** Schedule of enrollment, interventions, and assessments.

Action	Anesthesia	Preanesthetic visit	Presurgical visit	During surgery
Location	Consultation office	Hospital room	Specific room	Operation room
Timeliness	(−50 days; −5 days)	(−1 day)	(d-day)^b^	(d-day)
Consent collection	N/A^a^	N/A	N/A	N/A
Consent confirmation	N/A	N/A	✓	N/A
Providing information documents	✓	N/A	N/A	N/A
Checking inclusion and exclusion criteria	✓	N/A	✓	N/A
Medical interview	✓	✓	✓	N/A
Clinical examination	✓	✓	✓	✓
Electroencephalographic recording	N/A	N/A	✓	✓
Electromyography recording	N/A	N/A	N/A	✓
Median nerve stimulation	N/A	N/A	✓	✓
Adverse event collection	N/A	N/A	N/A	✓

^a^N/A: not applicable.

^b^d-day: day of experimentation.

Table 1 shows the registration, intervention, and evaluation schedule.

Considering the SEP stimulation, we consider that nonpainful stimuli will consist of square electrical pulses of 0.2 milliseconds of duration, generated by Micromed device Sd Ltm Stim Energy (Micromed) and delivered through a pair of grass gold cup electrodes (cathode [−] placed proximally) to the right/left median nerve at the wrist [32]. The intensity will be adjusted for eliciting visible small thumb twitches and without exceeding 50 mA.

### Study Population

In this clinical protocol, a total of 30 patients who require scheduled surgery under general anesthesia will be enrolled. For the recruitment of this study, we have designed a flyer that will be disseminated through different media channels. Participation in this experiment will not be remunerated. Patients will be exposed to the same risks of general anesthesia limited to propofol. The doses used in this clinical protocol are the same as those that induce loss of consciousness during conventional surgery.

The inclusion criteria include patients (1) who have received full details of the research organization and have signed our informed consent, (2) aged between 18 and 81 years, (3) scheduled for surgery with the use of total intravenous sedation with propofol, and (4) who are affiliated to a social security regime in Belgium. We shall exclude patients who (1) are allergic to propofol or its ingredients, (2) have a history of an anaphylactic reaction during anesthesia, (3) are female due to the impossibility of checking pregnancy status, (4) are deprived of liberty by a judicial or administrative decision, (5) are under psychiatric care, (6) are unable to give consent and without being subjected to a legal protection measure, (7) refuse to participate in the study, (8) have a BMI below 20 or above 35 kg/m², (9) require surgery for less than an hour, (10) have a pathological history related to the right median nerve, and (11) have any drug addiction.

### Ethical Considerations

This research was officially approved by an ethical committee from Belgium (CHU Brugmann, CE 2021/225) and was registered at EUDRACT (2021-006457-56). The study protocol was also registered on ClinicalTrials.gov (NCT05272202). This protocol will follow the principles of the Declaration of Helsinki and the Medical Research Involving Human Subjects Act [33]. Informed written consent will be obtained from all patients before study inclusion. Because involvement of patients is voluntary, the study can be stopped at any time.

### EEG Data Acquisition

Motor EEG signals will be collected through the OpenViBE software platform with eego mylab system (ANT neuro) 64-EEG channels, covering the entire scalp at 16,000 Hz and 1 external channel placed on the dorsum of the contralateral hand to the electric stimulation, by means of an adhesive surface electrode. All offline analyses will be performed using the EEGLAB toolbox [34] and Matlab2016a (The MathWorks Inc). The findings will be additionally investigated by applying a Laplacian filter and a mastoidal re-referencing [35]. Then, the EEG signals will be resampled (128 Hz) and epoched into 6-s windows (1 second before and 5 seconds after the MNS). We will compute the ERD/ERS% using the “band power method” [36]. Additionally, SEP will be calculated by averaging epochs extracted from 0.25 to 0.5 with respect to the electric shock.

### Statistics

The data processing methods will be similar to those described in the MOTANA protocol [23]. For each patient and each MNS, the maximal value of ERD and ERS max will be, respectively, selected in (0.25;0.5) seconds and (2;4) seconds after stimulation, in accordance with the literature [24,37]. An average over the ERDs max and ERSs max will be performed for each concentration. These 2 values will be, respectively, compared with average ERDs max and ERSs max obtained in the preoperative rest condition with a Student *t* test (*P*<.05). To visualize the event-related spectral perturbations with the EEGLAB toolbox, a surrogate permutation test will be used (*P*<.05; 2000 permutations). In addition to the permutation test, we will apply a false discovery rate correction to show the difference in MNS effect during different concentrations of propofol.

In addition to the event-related spectral perturbation analyses, we will apply 4 different offline machine learning algorithms to detect the MNS at the cerebral level. To do this, these algorithms are based on 4 classification methods in a 4-fold cross-validation scheme. The classification score will be computed for the MNS versus the rest class. Each trial will be segmented into a motor task time window and a resting time window, both lasting 2 seconds. The motor task time window will begin 0.5 seconds after the MNS, and the resting phase time window will begin 3 seconds before the MNS. The first classification method is a linear discriminant analysis method using a common spatial pattern [38] (referred to as common spatial pattern+linear discriminant analysis). For the next 3 classifiers, we will use classifiers that are based on Riemannian geometry. Riemannian geometry-based methods have the advantage of being immune to linear transformations and to be particularly efficient for the detection of motor intentions. For the statistical differences in the latency and topographic voltage of the SEP components, similar permutation tests and post hoc tests will be applied.

### Study Duration

The duration of the participation is from 45 minutes before surgery (installation of the EEG cam and the median nerve stimulator) until the end of surgery.

The expected overall duration of the research, including the time required for the completion of the data analysis, is 4 years. The promoter (CHU Brugmann) reserves the right to stop the research (1) if serious, there are significant procedural deviations in the protocol that could impact the statistical analysis of the data, (2) if recruitment of subjects is not adequate, or (3) if a significant issue regarding the safety and rights of the subjects arises.

### Data Collection

Data will be collected with a case report form and patient data will be anonymized (ie, a unique study number will be assigned to each individual). The case report form will contain demographic information of the patient, undesirable events which may be experienced during the study, the anesthesia chart will be photocopied at the end of the procedure and anonymized, and EEG data stored electronically under the supervision of the principal investigator.

### Patient and Public Involvement

A total of 30 patients will be involved in the STIM-MOTANA protocol. Patients, patient advisors, and the public were not involved in the development of the research questions or in the design of the study. Patient involvement in the study includes answering survey questionnaires at baseline and at each follow-up. Summaries of study results will be disseminated to study participants via email or mail. The results will be published in international peer-reviewed journals, and summaries will be provided to the funders and patients.

## Results

### Evaluation Criteria

#### Primary End Points

A total of 30 patients will be involved in the STIM-MOTANA protocol. We will compute the amplitude of ERD/ERS modulations before and after each MNS during the different phases of anesthesia at 2 different times: when the patient is awake before surgery and under general anesthesia. The primary evaluation end point will be the amplitude of the ERD. According to the Pfurtscheller method, ERD/ERS modulations will be calculated using a baseline taken before each stimulation [36]. The changes in ERD/ERS during MNS according to the various propofol concentrations will be continuously monitored. To compare the motor EEG signal before and after injection of the anesthetic product, we will apply paired statistical tests (Student *t* test).

#### Secondary End Points

In this project, we need to reach the highest MI EEG detection accuracies as possible, which is necessary in the context of a surgery for which we need a very high true positive rate (ideally all AAGAs are detected) as well as a very low false positive rate (ideally no false alarm). To do so, we will build BCI classifiers based on Riemannian geometry [22,24] a framework in which EEG signals are represented as covariance matrices, which are directly analyzed and classified.

#### Trial Status

When the manuscript was initially submitted, recruitment had not started. The recruitment began in December 2022. The current protocol version is version 1 and was previously accepted by the ethical committee of the CHRU Brugmann Hospital.

## Discussion

### Principal Findings

STIM-MOTANA is an interventional study aimed at designing an innovative BCI-based EEG-motor brain activity, which would detect the intention to move of a patient during general anesthesia. STIM-MOTANA is the first study to investigate cerebral motor activity modulations following peripheral nerve stimulation during general anesthesia.

AAGA can occur unexpectedly and is therefore a complication that cannot be easily anticipated and that is only retrospectively diagnosed. The STIM-MOTANA protocol proposes to detect intraoperative awareness reliably by analyzing, in real-time, brain motor activity under general anesthesia with a BCI based on MNS and new machine learning methods. Designing such a BCI presents us with 2 challenges. The first one is to detect the MI of a person who is a victim of AAGA without any time marker presented to the subject (ie, asynchronously) since, during AAGA, it is impossible to ask and know exactly when the patient will intend to move. Unfortunately, the literature clearly shows insufficient classification accuracies and high false positive rates with asynchronous BCI [22]. The second challenge is, therefore, to obtain a high level of accuracy (>90%—the minimum required accuracy according to the clinical application) to ensure a reliable BCI device that can be used in hospitals. To satisfy these 2 requirements, our objective is to design a robust MNS-based BCI. Based on strong practical and theoretical scientific reasoning from our previous studies, our innovative MNS-based BCI would provide a way to detect intraoperative awareness during general anesthesia. The primary limitation of this study is that ERD/ERS habitually generated by media nerve stimulation without anesthetic may disappear for deep levels of anesthesia.

### Conclusions

STIM-MOTANA will provide the first protocol study to investigate MNS cerebral motor effect during general anesthesia for the detection of intraoperative awareness. Based on strong practical and theoretical scientific reasoning from our previous studies, an innovative MNS-based BCI used in this study would provide a way to detect intraoperative awareness during general anesthesia.

## Appendix

Multimedia Appendix 1 Peer review report by Fonds de la Recherche Scientifique (FNRS) / National Funds of Research of Belgium - SVS-3 Sciences de la Vie et de la Santé - 3 (Brussels, Belgium).

## Abbreviations

AAGA: accidental awareness during general anesthesia
BCI: brain-computer interface
EEG: electroencephalography
ERD: event-related desynchronization
ERS: event-related synchronization
MIL: movement intention
MNS: median nerve stimulation
SEP: somatosensory evoked potential
TCI: target-controlled infusion

## Data Availability

The datasets generated and analyzed during the current study will be available from the corresponding author on request. Records of all patients will be kept separately in a secure place in the CHU-Brugmann.

## References

[1] Tasbihgou SR, Vogels MF, Absalom AR (2018). Accidental awareness during general anaesthesia: a narrative review. Anaesthesia.

[2] Pandit JJ, Andrade J, Bogod DG, Hitchman JM, Jonker WR, Lucas N, Mackay JH, Nimmo AF, O'Connor KO, O'Sullivan EPO, Paul RG, Palmer JHMG, Plaat F, Radcliffe JJ, Sury MRJ, Torevell HE, Wang M, Hainsworth J, Cook TM, Royal College of Anaesthetists, Association of Anaesthetists of Great Britain and Ireland (2014). 5th National Audit Project (NAP5) on accidental awareness during general anaesthesia: summary of main findings and risk factors. Br J Anaesth.

[3] Macgregor K (2013). A waking nightmare: how can we avoid accidental awareness during general anaesthesia?. J Perioper Pract.

[4] Pandit JJ, Cook TM (2013). National Institute for Clinical Excellence Guidance on measuring depth of anaesthesia: limitations of EEG-based technology. Br J Anaesth.

[5] Liu X, Lauer KK, Ward BD, Li SJ, Hudetz AG (2013). Differential effects of deep sedation with propofol on the specific and nonspecific thalamocortical systems: a functional magnetic resonance imaging study. Anesthesiology.

[6] Sebel PS, Bowdle TA, Ghoneim MM, Rampil IJ, Padilla RE, Gan TJ, Domino KB (2004). The incidence of awareness during anesthesia: a multicenter United States study. Anesthe Analg.

[7] Weiser TG, Haynes AB, Molina G, Lipsitz SR, Esquivel MM, Uribe-Leitz T, Fu R, Azad T, Chao TE, Berry WR, Gawande AA (2016). Size and distribution of the global volume of surgery in 2012. Bull World Health Organ.

[8] Weiser TG, Regenbogen SE, Thompson KD, Haynes AB, Lipsitz SR, Berry WR, Gawande AA (2008). An estimation of the global volume of surgery: a modelling strategy based on available data. Lancet.

[9] Bischoff P, Rundshagen I (2011). Awareness under general anesthesia. Dtsch Arztebl Int.

[10] Leslie K, Chan M, Myles PS, Forbes A, McCulloch TJ (2010). Posttraumatic stress disorder in aware patients from the B-aware trial. Anesth Analg.

[11] Xu L, Wu A, Yue Y (2009). The incidence of intra-operative awareness during general anesthesia in China: a multi-center observational study. Acta Anaesthesiol Scand.

[12] Kent CD, Domino KB (2009). Depth of anesthesia. Curr opin Anaesthesiol.

[13] Gao WW, He YH, Liu L, Yuan Q, Wang YF, Zhao B (2018). BIS monitoring on intraoperative awareness: a meta-analysis. Curr Med Sci.

[14] Lewis SR, Pritchard MW, Fawcett LJ, Punjasawadwong Y (2019). Bispectral index for improving intraoperative awareness and early postoperative recovery in adults. Cochrane Database Syst Rev.

[15] Mashour GA, Avidan MS (2015). Intraoperative awareness: controversies and non-controversies. Br J Anaesth.

[16] Messina AG, Wang MW, Ward MJ, Wilker CC, Smith BB, Vezina DP, Pace NL (2016). Anaesthetic interventions for prevention of awareness during surgery. Cochrane Database Syst Rev.

[17] Ghoneim MM, Weiskopf RB (2000). Awareness during anesthesia. Anesthesiology.

[18] Lotte F, Congedo M (2016). Brain–computer interfaces 1: foundations and methods. EEG Feature Extraction.

[19] Cheron G, Cebolla AM, De Saedeleer C, Bengoetxea A, Leurs F, Leroy A, Dan B (2007). Pure phase-locking of beta/gamma oscillation contributes to the N30 frontal component of somatosensory evoked potentials. BMC Neurosci.

[20] Cebolla AM, De Saedeleer C, Bengoetxea A, Leurs F, Balestra C, d'Alcantara P, Palmero-Soler E, Dan B, Cheron G (2009). Movement gating of beta/gamma oscillations involved in the N30 somatosensory evoked potential. Hum Brain Mapp.

[21] Neuper C, Wortz M, Pfurtscheller G (2006). ERD/ERS patterns reflecting sensorimotor activation and deactivation. Prog Brain Res.

[22] Rimbert S, Riff P, Gayraud N, Schmartz D, Bougrain L (2019). Median nerve stimulation based BCI: a new approach to detect intraoperative awareness during general anesthesia. Front Neurosci.

[23] Rimbert S, Schmartz D, Bougrain L, Meistelman C, Baumann C, Guerci P (2019). MOTANA: study protocol to investigate motor cerebral activity during a propofol sedation. Trials.

[24] Rimbert S, Guerci P, Gayraud N, Meistelman C, Bougrain L (2019). Innovative brain-computer interface based on motor cortex activity to detect accidental awareness during general anesthesia.

[25] Avilov O, Rimbert S, Popov A, Bougrain L (2021). Optimizing motor intention detection with deep learning: towards management of intraoperative awareness. IEEE Trans Biomed Eng.

[26] Guerci P, Bougrain L, Schmartz D, Losser M, Meistelman C, Rimbert S (2021). Effet d'une sédation légère au propofol sur les oscillations cérébrales du cortex moteur: étude randomisée, contrôlée chez le volontaire sain. https://hal.univ-lorraine.fr/hal-03265548.

[27] Mashour GA, Orser BA, Avidan MS, Warner DS (2011). Intraoperative awareness: from neurobiology to clinical practice. Anesthesiology.

[28] Peltier SJ, Kerssens C, Hamann SB, Sebel PS, Byas-Smith M, Hu X (2005). Functional connectivity changes with concentration of sevoflurane anesthesia. Neuroreport.

[29] Schnider TW, Minto CF, Gambus PL, Andresen C, Goodale DB, Shafer SL, Youngs EJ (1998). The influence of method of administration and covariates on the pharmacokinetics of propofol in adult volunteers. Anesthesiology.

[30] Schnider TW, Minto CF, Shafer SL, Gambus PL, Andresen C, Goodale DB, Youngs EJ (1999). The influence of age on propofol pharmacodynamics. Anesthesiology.

[31] Absalom AR, Mani V, De Smet T, Struys MMRF (2009). Pharmacokinetic models for propofol--defining and illuminating the devil in the detail. Br J Anaesth.

[32] Schnitzler AS, Salenius S, Salmelin R, Jousmäki V, Hari R (1997). Involvement of primary motor cortex in motor imagery: a neuromagnetic study. Neuroimage.

[33] World Medical Association (2002). World medical association declaration of helsinki: ethical principles for medical research involving human subjects. J Postgrad Med.

[34] Delorme A, Makeig S (2004). EEGLAB: an open source toolbox for analysis of single-trial EEG dynamics including independent component analysis. J Neurosci Methods.

[35] Perrin F, Pernier J, Bertrand O, Echallier JF (1989). Spherical splines for scalp potential and current density mapping. Electroencephalogr Clin Neurophysiol.

[36] Pfurtscheller G, Lopes da Silva F (1999). Event-related EEG/MEG synchronization and desynchronization: basic principles. Clin Neurophysiol.

[37] Salenius S, Schnitzler A, Salmelin R, Jousmäki V, Hari R (1997). Modulation of human cortical rolandic rhythms during natural sensorimotor tasks. Neuroimage.

[38] Blankertz B, Tomioka R, Lemm S, Kawanabe M, Muller KR (2008). Optimizing spatial filters for robust EEG single-trial analysis. IEEE Signal Process Mag.

