# Supplementary material for: Detection of Motor Cerebral Activity After Median Nerve Stimulation During General Anesthesia (STIM-MOTANA): Protocol for a Prospective Interventional Study
Source: JMIR Res Protoc. 2023 Feb 2;12:e43870. doi: 10.2196/43870 (PMC10013682; doi:10.2196/43870)
Supplement: Multimedia Appendix 1 [file resprot_v12i1e43870_app1.pdf]

Fonds de la Recherche Scientifique - FNRS

EVALUATION FINAL REPORT / RAPPORT FINAL D'ÉVALUATION

Appel/Call "Bourses et Mandats/Grants and Fellowships" 2021

REFERENCES OF THE APPLICATION / REFERENCES DE LA DEMANDE

Chargé de recherches [CR]

Commission : SVS-3 Sciences de la Vie et de la Santé - 3

Application ID / N° de la demande : 40005428

Host institution / Institution d'accueil : Université libre de Bruxelles

Participants :
RIMBERT Sébastien [Applicant / Proposant]
CEBOLLA Ana-Maria (U.L.B.) [Promoter / Promoteur]

Titre de la proposition :
Une Interface Cerveau-Ordinateur innovante pour détecter le réveil peropératoire pendant l'anesthésie générale

Proposal title:
An innovative Brain-Computer Interface to detect intraoperative awareness during general anesthesia

EVALUATION FINAL REPORT / RAPPORT FINAL D'ÉVALUATION

Commission report / Rapport de la Commission

Criterion : The researcher / Critère : Le chercheur

Sébastien Rimbert is an ambitious and competent scientist with promising skills for conducting innovative research in the field of BCI and related medical applications. His interdisciplinary formation will be very useful to the research he proposes to develop.

Criterion : The research project / Critère : le projet de recherche

The overall project is original and ambitious. The already synergistic interaction between the applicant and partners and their expertise allows for a feasible research plan whose final achievements are however of high-risk and no alternative plan is provided. Yet, even if the main goal may not be achieved, there will be still "sub"-outcomes relevant to different fields such as machine learning, BCI, and EEG pattern recognition.

Criterion : The research environment / Critère : L'environnement de recherche

The applicant has already built up the multidisciplinary network - including the respective facilities - to conduct the proposed research in an outstanding environment

Final comment / Commentaire final

Ambitious and high-risk project submitted by a promising young scientist in a perfectly adequate environment.

Commission final grading / Note finale donnée par la Commission

A : Excellent / A : Excellent

DECISION OF THE BOARD OF TRUSTEES / DÉCISION DU CONSEIL D'ADMINISTRATION

Granting / Octroi

LA LIBERTÉ DE CHERCHER

REMOTE EVALUATION REPORTS (1ST STEP) / RAPPORTS D'ÉVALUATION À DISTANCE (ETAPE 1)

[Review 114708]

Criterion : The researcher / Critère : Le chercheur

Strengths / Forces

The candidate has been working on brain-computer interfaces (BCI) for 6 years. He has an interdisciplinary formation (cognitive sciences, computer science, psychology, and neuroscience). He has worked on BCIs based on Motor Imagery (MI), mainly to extend the understanding of these BCIs, and to improve their efficiency, exploring different motor imaging tasks, different feedback and user training protocols. He also has contributed to the creation of new algorithms for the analysis and classification (machine learning) of EEG signals. Regarding clinical research, the candidate has strong experience in this field, both in general anesthesia, stroke rehabilitation, and hypnosis. During his PhD and post-doctorate, he has co-authored 10 studies involving more than 120 participants, publishing more than 25 articles (including 6 journal articles) with a total of 85 citations.

Weaknesses / Faiblesses

He has done few international mobilities, although he has participated in some international conferences (China, Japan, USA, Wales).

General comment / Commentaire général

He has a very strong experience in MI-BCIs, in addition to having an interdisciplinary formation (cognitive sciences, computer science, psychology, and neuroscience), which will be very useful to the research he proposes to develop.

Criterion : The research project / Critère : le projet de recherche

Strengths / Forces

This proposal aims to develop an innovative MI-BCI to accurately detect the intentions of movement of patients during an AAGA (Accidental Awareness during a General Anesthesia). Specifically, the research has as a innovative goal to detect intraoperative awareness reliably by analyzing, in real-time, brain motor activity under general anesthesia with a BCI based on Median Nerve Stimulation (MNS) and innovative machine learning methods.

Weaknesses / Faiblesses

There is no evidence that movement intention induces detectable changes in EEG patterns with increasing doses of anesthetics even when using MNS.

It will be necessary to develop a highly accurate BCI with a very low false-positive rate

Before a real surgery, medical doctors do not have time to collect samples of EEG signals from patients during MI, which are necessary to label EEG
data resulting from movement intention and thus calibrating the BCI to detect MI during general anesthesia

General comment / Commentaire général

Although several chanlenges must be faced by the candidate, the research proposal is solid and the candidate has quite skill to make it.

Criterion : The research environment / Critère : L'environnement de recherche

Strengths / Forces

This research proposal will be carried out at the Laboratory of Neurophysiology and Movement Biomechanics (LNMB, Université Libre de Bruxelles), which has an international expertise in the area of EEG, evoked potentials, movement analysis and BCI. The LNMB's equipment provides the possibility to record simultaneously EEG, ERP, EMG and 3D motion in a real environment. In addition, ANT high-density (128-l and 64 channels) EEG systems are used in parallel with the optoelectronic VICON system (10 cameras working at 100 Hz) for motion capture and synchronized to the 16 wireless electromyographic channels (DELSYS) and the Eye-tracker system (TOBII) are available to this research. Also, the LNMB guarantees all the necessary conditions (intellectual, material, and human) to ensure the success of this research.

It is worth commenting that this research relies on a top-level interdisciplinary consortium, combining high expertise in the fields of the medical practice of General Anesthesia (CHU Brugmann, D. Schmartz & S. Rimbert), Neurosciences and Cerebral Motor activity (Laboratory of Neurophysiology and

Movement Biomechanics (LNMB), ULB, A. Cebolla & G. Cheron), MNS-based BCI (S. Rimbert) and Riemannian Geometry and Machine Learning Algorithms for BCI (G. Cheron, S. Rimbert and A. Cebolla). All partners have already collaborated with one or two other members, which will facilitate synergies.

Weaknesses / Faiblesses

No detected

General comment / Commentaire général

The Laboratory of Neurophysiology and Movement Biomechanics (LNMB, Université Libre de Bruxelles) has an international expertise in the area of EEG, evoked potentials, movement analysis and BCI, and has the suitable equipment necessary to this research. In addition, this research this relies on a top-level interdisciplinary consortium, combining high expertise in the fields of the medical practice of General Anesthesia, Neurosciences and Cerebral Motor activity , MNS-based BCI and Riemannian Geometry and Machine Learning Algorithms for BCIs.

Global comment / Commentaire global

The candidate has been working on brain-computer interfaces (BCI) for 6 years, and has an interdisciplinary formation (cognitive sciences, computer science, psychology, and neuroscience). He has worked on several aspects of BCIs based on Motor Imagery (MI). Regarding clinical research, the candidate has strong experience in this field, both in general anesthesia, stroke rehabilitation, and hypnosis. During his PhD and post-doctorate, he has co-authored several studies involving more than 120 participants and published more than 25 articles (including 6 journal articles) with a total of 85 citations, which demonstrates his expertise in this research field, implying in a high degree of expected success for this research proposal aiming to detect intraoperative awareness reliably by analyzing, in real-time, brain motor activity under general anesthesia with a BCI based on Median Nerve Stimulation (MNS) and innovative machine learning methods.

In additon, the Laboratory of Neurophysiology and Movement Biomechanics (LNMB, Université Libre de Bruxelles) has an international expertise in the area of EEG, evoked potentials, movement analysis and

BCI, and has the suitable equipment necessary to this research. A top-level interdisciplinary consortium will part of this research, which has high expertise in the fields of the medical practice of General Anesthesia, Neurosciences and Cerebral Motor activity , MNS-based BCI and Riemannian Geometry and Machine Learning Algorithms for BCIs.

Ethical issues / Aspects éthiques

The ethical aspects of a proposal must be described, as well as the way in which the applicant(s) plan(s) to treat them. Examples of potential ethical issues use and storage of private data, handling of substances potentially damageable to the environment, research involving animals or human beings. / Les aspects éthiques d'une proposition doivent être décrits, de même que la manière dont le(s) proposant(s) prévoi(en)t de les traiter. Exemples de problèmes éthiques potentiels : utilisation et stockage de données privées, manipulation de substances pouvant créer des dommages à l'environnement, recherche sur des animaux ou des êtres humains.

Has(have) the applicant(s) taken into consideration the ethical aspects in his/her (their) proposal ? Indicate "irrelevant" if need be. / Le(s) proposant(s) a-t-il (ont-ils) pris en compte les aspects éthiques dans son (leur) projet ? Indiquez "non applicable" le cas échéant.

Yes / Oui

Indicate an optional comment regarding the ethical aspects of the proposal. / Indiquez un commentaire optionnel relatif aux aspects éthiques de la proposition.

No comment / Pas de commentaire

[Review 114682]

Criterion : The researcher / Critère : Le chercheur

Strengths / Forces

Dr. Rimbert is an interdisciplinary postdoctoral researcher very experienced in the field of median nerve stimulation and motor cortical excitability as well as machine learning. He, thus, combines two aspects of expertise mandatory to execute the proposed research: profound neuroscientific knowledge and potential to apply machine learning approaches to new fields and scientific challenges. I watched Dr. Rimberts PhD defense and was quite impressed how professionally he presented his results, very structured, logically set-up and very self-confidently. He actively publishes his results and is interested and knowledgable in adjacent field important for his research, such as aspects linked to deep sedation for surgery and ethical aspects of his research. His CV demonstrates that he is also active in para-scientific activities such as promotion of young researchers.

Weaknesses / Faiblesses

I admit, those are difficult to find and I had to search for one. The only weakness I came across was, that in his academic record, his PhD thesis is the only one which received a "très honorable". However, I would interpret this as a sign that if Dr. Rimbert can work for goals he is fully interested in, allows him to develop and unfold his full potential. Also, I am not fully aware of the French grading system, thus, I do not know the exact differences between "très honorable" and "assez bien".

General comment / Commentaire général

I consider Dr. Sébstian Rimbert an ambitious and competent researcher with great potential for contributing significant results to the fields of neuroscience and BCI.

Criterion : The research project / Critère : le projet de recherche

Strengths / Forces

- tackles into a clinical very relevant field, i.e. Accidental Awareness during a General Anesthesia (AGAA), which can lead to posttraumatic stress disorder.

- sound background work has been already carried out, thus, solid scientific basis for the proposed work

- inclusion of healthy participants and patients in need of surgery; large samples thereof

- combination of solid neuroscientific knowledge, such as motor excitability and pathways with brain-computer interfacing and machine learning - ...

-...multidisciplinary reasearch proposal, for which the applicant covers several aspects himself: machine learning, BCI, nerve stimulation

- combination of basic and applied research

- logically set-up work packages

- contribution to the field of BCI and EEG pattern detection of unlabelled data independent of the direct success of the project with respect to the detection of AAGA

- systematic research on the effect of different doses of propofol on EEG patterns elicited by median nerve stimulation and motor imagery

- development of new algorithms potentially suitable to detect motor imagery pattern in unlabeled data, which are encountered under real world conditions of patients becoming aware under anesthesia; potentially transferable to other classification problems

Weaknesses / Faiblesses

The project is very ambitious and thus, has the potential to not being able to fully accomplish all the tasks. Yet, this is inherent for a project tackling into an unknown field.

General comment / Commentaire général

A high risk project, with a sound background and logical set-up time- and research-wise. Interdisciplinary expertise on board and confirmed committment of the respective people. If successful the clinical contribution of the project would be enormous. Thus, the high risk may lead to high scientific and clinical outcome. Apart from this very clinical and applied scientific aspect, the project will also contribute to a better understand the effects of anesthesia on motor cortical excitability and will provide algorithms for classification of unlabelled data. Thus, even if the main goal may not be achieved, there will be still "sub"-outcomes relevant to different fields such as machine learning, BCI, and EEG pattern recognition.

The project is ambitious as is the resarcher!

Criterion : The research environment / Critère : L'environnement de recherche

Strengths / Forces

- All facilities necessary for the project available (EEG Lab, access to patients, possibility of experimentation with propofol)
- international collaboration with researchers in the different fields already set-up and...
- ...explicit committment of those demonstrated

Weaknesses / Faiblesses

None

General comment / Commentaire général

The applicant has already built up the multidisciplinary network - including the respective facilities - to conduct the proposed research

Global comment / Commentaire global

I rate the applicant as highly qualified and capable of conducting the proposed research as it builts on solid ground: with his PhD and publications he demonstrated his knowledge and also practical expertise in the respective field.

The project is highly relevant, scientifically thought through, challenging and potentially groundbreaking.

Ethical issues / Aspects éthiques

The ethical aspects of a proposal must be described, as well as the way in which the applicant(s) plan(s) to treat them. Examples of potential ethical issues use and storage of private data, handling of substances potentially damageable to the environment, research involving animals or human beings. /

Les aspects éthiques d'une proposition doivent être décrits, de même que la manière dont le(s) proposant(s) prévoi(en)t de les traiter. Exemples de problèmes éthiques potentiels : utilisation et stockage de données privées, manipulation de substances pouvant créer des dommages à l'environnement, recherche sur des animaux ou des êtres humains.

Has(have) the applicant(s) taken into consideration the ethical aspects in his/her (their) proposal ? Indicate "irrelevant" if need be. / Le(s) proposant(s) a-t-il (ont-ils) pris en compte les aspects éthiques dans son (leur) projet ? Indiquez "non applicable" le cas échéant.

Yes / Oui

Indicate an optional comment regarding the ethical aspects of the proposal. / Indiquez un commentaire optionnel relatif aux aspects éthiques de la proposition.

No comment / Pas de commentaire

[Review 115366]

Criterion : The researcher / Critère : Le chercheur

Strengths / Forces

The researcher (PI) is an active young researcher/scientist in the field of neuroscience -specifically of BCI/BMI. He demonstrates very good educational track with training in engineering science, informatic and life science. He also demonstrates an active and promising experience in attending different research labs at national and international levels. Along his young career, there are several past and present collaborations within projects which are relevant to the application project. Very good track of publications, although in specialty journals. Very good track of grants as a young investigator.

Weaknesses / Faiblesses

Track of publications is good with good citation track, but they are only in specialty journals. One recent award in specialty session of international conference.

General comment / Commentaire général

Overall, the research applicant demonstrates promising skills in conduction innovative research in the field of BCI/BMI for medical applications. The portfolio of publications still awaits for major achievements in journals with a broader audience.

Criterion : The research project / Critère : le projet de recherche

Strengths / Forces

The project concerns the design and develop a novel class of EEG-based BCI for a real-time detection of intention to move under human intraoperative general anethesia. The rationale behind such research plan is that no accurate detection procedure is currently available to prevent AAGA (Accidental Awareness during General Anesthesia) which can occur up to 2% of cases (estimation in France). The project is based on previous findings/assumption that the Median nerve stimulation associated to intention to move determines a change in the EEG signature (ERD/ERS) of the motor intention/imagery such that it can be better detected by machine learning approaches. As such the idea of a “passive” EEG-BCI monitoring the level of awareness to ultimately detect in real-time its variation during surgery general anesthesia is substantially original as it is the target medical application. Conceptually, this is an ambitious project as well as of high risk with several unknowns (see below). On the other hand, the project may have potential scientific and industrial gain (impact) if major parts of it turn out successful.

The work plan is well-structures in interactive 4 WPs within an adequate time frame. The interdisciplinarity and solid expertise of the partners/promoter (medicine-general anesthesia; machine learning; brain motor function; BCI) guarantees the feasibility of the research plan, including the 2 planned clinical studies (CT1 and 2) aiming at collecting data on motor oscillatory activity under different doses of anesthetics (ie propofol) in healthy and patient sample. A communication plan is provided for project results exploitation.

Weaknesses / Faiblesses

The weaknesses of the research plan mainly reside in the lack of a risk assessment (which is at least to be mentioned in an ambitious project that aims at implementing a >90% accuracy, no calibration EEG-based BCI to detect motor intention under anesthesia) and thus, of a mitigation/alternative plan. What if, the model of EEG motor signature under anesthesia to be generated based on multidimension

parameters will not be transferable in real-time? How much would be the trade-off between real-time detection and accuracy that can be considered as acceptable?

General comment / Commentaire général

The overall project is original and ambitious. The already synergistic interaction between the applicant and partners and their expertise allows for a feasible research plan whose final achievements are however of high risk and no alternative plan is provided.

Criterion : The research environment / Critère : L'environnement de recherche

Strengths / Forces

The research laboratory infrastructures (both the applicant lab and the partner at INRIA Bordeaux), and the medical unit (Belgium) are highly qualified for the project aims. The scientific expertise of the project promoter (director of the research lab) and the overall project team is of high-quality standard.

Weaknesses / Faiblesses

NA

General comment / Commentaire général

The research environment in terms of knowledge/expertise, equipment and collaborative network where the research will be carried out is excellent.

Global comment / Commentaire global

The overall application is well-written and clear in its challenges/objectives.

The PIs CVs are very good with an excellent environment where the research project will be carried out. The overall project is original and ambitious as well as of high risk especially concerning the ultimate goal of implementing a >90% accurate no calibration passive EEG-based BCI for online detection of awareness fluctuation under general anesthesia in humans.

Ethical issues / Aspects éthiques

The ethical aspects of a proposal must be described, as well as the way in which the applicant(s) plan(s) to treat them. Examples of potential ethical issues use and storage of private data, handling of substances potentially damageable to the environment, research involving animals or human beings. / Les aspects éthiques d'une proposition doivent être décrits, de même que la manière dont le(s) proposant(s) prévoi(en)t de les traiter. Exemples de problèmes éthiques potentiels : utilisation et stockage de données privées, manipulation de substances pouvant créer des dommages à l'environnement, recherche sur des animaux ou des êtres humains.

Has(have) the applicant(s) taken into consideration the ethical aspects in his/her (their) proposal ? Indicate "irrelevant" if need be. / Le(s) proposant(s) a-t-il (ont-ils) pris en compte les aspects éthiques dans son (leur) projet ? Indiquez "non applicable" le cas échéant.

Yes / Oui

Indicate an optional comment regarding the ethical aspects of the proposal. / Indiquez un commentaire optionnel relatif aux aspects éthiques de la proposition.

no data protection information available
